# Clinical and genetic characterization of intellectual disability

**DOI:** 10.1111/dmcn.70252

**Published:** 2026-03-17

**Authors:** Aarni Venetvaara, Minna Kraatari‐Tiri, Jussi‐Pekka Tolonen, Marko Merikukka, IDGEN Study Group, Hannaleena Kokkonen, Teija Paakkola, Susanna Varis, Antti Dahl, Jarmo Körkkö, Jonna Komulainen‐Ebrahim, Jukka Moilanen, Johanna Uusimaa, Outi Kuismin, Elisa Rahikkala

**Affiliations:** ^1^ Clinical Genetics Unit Oulu University Hospital Oulu Finland; ^2^ Research Unit of Clinical Medicine and Medical Research Center Oulu Oulu University Hospital and University of Oulu Oulu Finland; ^3^ Pediatric Neurology Unit Oulu University Hospital Oulu Finland; ^4^ Research Service Unit, Research Unit of Clinical Medicine Oulu University Hospital Oulu Finland; ^5^ Northern Finland Laboratory Centre, NordLab Oulu Finland; ^6^ Intellectual Disabilities Unit Oulu University Hospital Oulu Finland; ^7^ Genomics Unit, Laboratory Division Turku University Hospital Turku Finland

## Abstract

**Aim:**

To examine the clinical and genetic characteristics of intellectual disability.

**Method:**

We conducted a population‐based retrospective analysis on the clinical and genetic data of 959 children with diagnosed intellectual disability during a 5‐year period (2017–2021) at Oulu University Hospital, Finland.

**Results:**

Pathogenic or likely pathogenic gene variants were detected in 89 of 194 patients (46%) who underwent exome sequencing. Chromosomal abnormalities, including those with low penetrance, were observed in 106 of 530 patients (20%) who underwent chromosomal microarray testing. Chromosomal abnormalities and causative gene variants were more frequently identified in patients with moderate to profound intellectual disability than in those with mild intellectual disability; however, this difference was not significant in the diagnostic yield analysis. Epilepsy, congenital heart disease, hearing loss, ophthalmological abnormalities, and autism spectrum disorder were more common among patients with moderate to profound intellectual disability, whereas attention‐deficit/hyperactivity disorder was associated with mild intellectual disability. Chromosomal abnormalities were associated with congenital heart disease and hearing loss, while pathogenic gene variants were associated with epilepsy and ophthalmological abnormalities.

**Interpretation:**

Somatic comorbidities were more common in moderate to profound intellectual disability, whereas attention‐deficit/hyperactivity disorder was more frequent in mild intellectual disability.

## Graphical abstract

This study examines the etiological factors and comorbidities in a large cohort of Finnish patients with intellectual disability. Genetic causes—including chromosomal abnormalities and pathogenic gene variants—were more frequently identified in individuals with moderate to profound intellectual disability. Additionally, somatic comorbidities such as epilepsy, congenital heart disease, hearing loss, and ophthalmological abnormalities were more common in severe cases, whereas attention‐deficit/hyperactivity disorder was more often associated with mild intellectual disability.

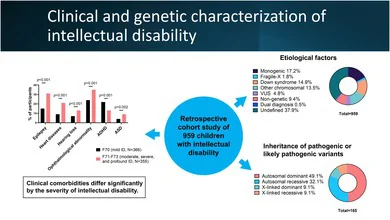

**This original article is commented by Hisama on pages** 1498–1499 **of this issue.**

**Abbreviations**
CHD: congenital heart disease
CMA: chromosomal microarray analysis

**What this paper adds**

- Clinical comorbidities differ by the severity of intellectual disability.
- Somatic comorbidities and autism are more prevalent in moderate to profound intellectual disability.
- Attention‐deficit/hyperactivity disorder is more frequently diagnosed in individuals with mild intellectual disability.
- Enrichment of population‐specific variants associated with intellectual disability was observed.
- Eleven per cent of patients with pathogenic variants had a potentially treatable aetiology.

Intellectual disability is a neurodevelopmental disorder affecting approximately 1% to 3% of the global population, and it imposes a significant economic burden on families, healthcare services, and societies.^1^ It can be caused by genetic or acquired factors, such as exposure to hazardous chemicals, infection during pregnancy, birth asphyxia, and childhood diseases such as herpes encephalitis or severe brain injury.^2^ The genetic aetiology of intellectual disability is highly heterogeneous, comprising various chromosomal abnormalities as well as pathogenic variants in single genes. It has been estimated that approximately 2000 genes are involved in intellectual disability.^3^

Genetic aetiologies have been studied more thoroughly in individuals with severe intellectual disability, while their significance in mild intellectual disability is less established. De novo pathogenic gene variants or chromosomal abnormalities are the most common cause of intellectual disability.^4^ The proportion of autosomal recessive variants causing intellectual disability increases in populations where consanguineous marriages are common.^5^, ^6^ A polygenic inheritance model has been suggested to explain at least part of mild intellectual disability.^6^, ^7^ Phenotypic spectra and causative genetic variants vary widely by ethnicity.

In this study, we delineate the clinical and genetic characteristics of intellectual disability in a population‐based unselected clinical cohort of patients with the disorder.

## METHOD

### Clinical data

The study population included children diagnosed with intellectual disability who visited Oulu University Hospital, Finland, over a 5‐year period (2017–2021). Inclusion criteria were patients younger than 18 years with a diagnosis of intellectual disability during the study period. The list of diagnoses in the International Classification of Diseases, 10th Revision (ICD‐10) included the group F70–F79 and aetiological diagnoses typically indicating intellectual disability (E72.5; E75.0,1,2,4,6; E76.0,1,2,3,8,9; E77.0,1,8,9; E79.1; E88.8; F84.2; Q00.0; Q03.81; Q04.00; Q04.2; Q04.51; Q86.0; Q87.01,05,12,15,18,19,23,30,31,38,83–86; Q90; Q91; Q92; Q93; and Q99.2) as described earlier.^8^ In total, there were 1160 children with any of the above‐mentioned ICD‐10 diagnoses during the study period. The presence of intellectual disability, especially in addition to the aetiological diagnoses, was confirmed by manual inspection of medical records. Individuals whose diagnosis of intellectual disability could not be confirmed by retrospective evaluation of clinical records (*n* = 154) and those living in other hospital districts with insufficient data available (*n* = 47) were excluded from the analysis, leaving a cohort of 959 patients with confirmed intellectual disability (Figure S1).

Clinical data included severity and aetiology of intellectual disability, genetic laboratory results, and the presence of additional clinical manifestations such as hearing loss, ophthalmological abnormalities, congenital heart disease (CHD), epilepsy, attention‐deficit/hyperactivity disorder (ADHD) (ICD‐10: F90.0), or autism spectrum disorder (ICD‐10: F84). These data were collected by retrospective review of electronic clinical records.

We also assessed whether there were possible acquired causes explaining the intellectual disability, such as known prenatal exposures, birth asphyxia, infections, preterm birth, or brain trauma. Preterm birth was assessed according to the classification of the World Health Organization.^9^

### Genetic testing and data analysis

Genetic testing was conducted from 2000 to 2022 as part of clinical diagnostics. The available tests varied by laboratory and by year of testing. Standard chromosome analysis was performed for 389 patients (*n* = 389 out of 959, 41%). Chromosomal microarray analysis (CMA) was performed in 530 patients (*n* = 530 out of 959, 55%). Exome sequencing and/or genome sequencing were programmed in 194 out of 959 (20%) and 11 out of 959 (1%) of the cohort respectively. Standard chromosome analysis and CMA were performed for 149 out of 959 (16%) of patients. CMA combined with exome sequencing was performed for 166 out of 959 (17%) of patients, and all three tests—standard chromosome analysis, CMA, and exome sequencing—were performed for 55 out of 959 (6%) of patients. A next‐generation sequencing‐based gene panel was performed for 25 out of 959 (3%) of patients. Sanger sequencing of whole genes was performed for 98 out of 959 (10%) of the cohort. Targeted testing of known familial variants was performed for 9 out of 959 (0.9%) of patients. Tests for fragile X repeat expansions, Prader–Willi syndrome, or Angelman methylation were performed in 382 out of 959 (40%), 30 out of 959 (3%), and 10 out of 959 (1%) of the cohort respectively, on the basis of clinical suspicion. Targeted fluorescence in situ hybridization was performed in 64 out of 959 (7%). Targeted testing of Finnish founder variants was performed in 61 out of 959 (6%) of patients on the basis of clinical suspicion.

The identified sequence variants were classified using criteria of the American College of Medical Genetics and Genomics.^10^ Pathogenic or likely pathogenic variants were considered clinically significant and included in the diagnostic yield analysis. Before 2020, when the guidelines of the American College of Medical Genetics and Genomics for structural variants were released,^11^ chromosomal abnormalities were not classified according to the modern criteria; reclassification of older reported structural variants was not performed.

Statistical significance between different subgroups was analysed using two‐tailed Fisher's exact and *χ* ^2^ tests. We used logistic regression analysis to assess whether severity of intellectual disability was associated with the likelihood of individuals having a pathogenic variant detected either in the CMA or in the exome sequencing test. Logistic regression was also used to assess whether the aetiology of intellectual disability was associated with the likelihood of comorbidities. Age and sex were used as covariates in both logistic regression analyses. All the statistical analyses were conducted using SPSS software, version 29 (IBM Corp., Armonk, NY, USA). Bonferroni correction was applied to account for multiple comparisons and Bonferroni corrected *p*‐values were calculated.

Variants' population frequencies were obtained from the Genome Aggregation Database (gnomAD version 4.1.0).^12^ Variants were considered Finnish‐enriched if the allele frequency in the Finnish population was at least threefold greater than any other population, or if the variant was present only in the Finnish population.

### Ethical approval

The study was performed according to the Declaration of Helsinki and approved by the Northern Ostrobothnia Hospital District (IDGEN 10/2022, accepted 19 May 2022). Written informed consents were not required for this study in accordance with national guidelines.

## RESULTS

### Clinical characteristics

The cohort included 959 patients: 560 males and 399 females. The mean age was 10 years 8 months (SD 5 years 6 month) and the median age was 10 (interquartile range 9) years. Demographic and clinical characteristics of the patients are summarized in Table 1, and Table 2 presents the associated phenotypes across aetiological categories. Notably, epilepsy was most common among patients with a non‐genetic aetiology (*n* = 49 out of 90, 54%).

**TABLE 1:** Demographic and clinical characteristics of the 959 patients evaluated in this study.

Characteristic	*n*	%
Sex		
Male	560	58
Female	399	42
Age (years)		
0–4	126	13
5–9	316	33
10–14	242	25
15–19	211	22
20+	64	7
Ethnicity		
Finnish^a^	928	97
Other	31	3
Severity of intellectual disability		
Mild	366	38
Moderate	175	18
Severe	120	13
Profound	60	6
Unspecified	238	25
Comorbidities^b^		
Epilepsy	254	26
Congenital heart diseases	137	14
Hearing loss	106	11
Ophthalmological abnormality	303	31
Any neuropsychiatric disorder		
Attention‐deficit/hyperactivity disorder	126	13
Autism spectrum disorder	54	7
**Total**	959	

^a^ Individual was considered Finnish if at least one of his parents was of Finnish ancestry.

^b^ Some individuals presented with multiple comorbidities.

**TABLE 2:** The number of patients with associated comorbidities in different aetiological categories.

	Down syndrome (*n* = 143)	Other chromosomal abnormalities (*n* = 129)	Pathogenic/likely pathogenic variants (*n* = 165)	Unknown (*n* = 410)	Non‐genetic aetiology (*n* = 90)
	*n* (%)	*n* (%)	*n* (%)	*n* (%)	*n* (%)
Epilepsy	11 (8)	33 (26)	69 (42)	89 (22)	49 (54)
CHDs	62 (43)	29 (23)	12 (7)	26 (6)	7 (8)
Hearing loss	33 (23)	18 (14)	18 (11)	20 (5)	13 (14)
Ophthalmological abnormality	43 (30)	47 (36)	80 (49)	89 (22)	40 (44)
ADHD	6 (4)	16 (12)	9 (6)	78 (19)	14 (16)
ASD	0 (0)	3 (2)	6 (4)	43 (11)	<3^a^ (<3)

*Note*: Some individuals presented with multiple comorbidities. Patients with fragile X syndrome are not included owing to the low number of individuals (*n* = 17).

Abbreviations: ADHD, attention‐deficit/hyperactivity disorder; ASD, autism spectrum disorder; CHDs, congenital heart diseases.

^a^ <3 means one or two individuals.

In the cohort with Down syndrome, 62 out of 143 (43%) had CHD; 33 out of 143 (23%) had hearing loss; 43 out of 143 (30%) had ophthalmological abnormalities such as strabismus; 11 out of 143 (8%) were diagnosed with epilepsy; and 6 out of 143 (4%) were diagnosed with an ADHD.

In total, 31 out of 959 (3%) members of the cohort were deceased. The most common aetiological diagnoses were non‐ketotic hyperglycaemia (E72.5, 11 cases), other intellectual disability causing inherited syndromes (six cases) and Down syndrome (Q90.0, four cases). Other diagnoses were trisomies Q91 (three cases), brain malformations (three cases), and other causes (idiopathic causes, traumas, and unknown, four cases).

### Genetic results

Overall, pathogenic or likely pathogenic gene variants or chromosome abnormalities underlying the intellectual disability were identified in 437 out of 959 (46%) of patients (Figure 1a and Table S1).

**FIGURE 1:**
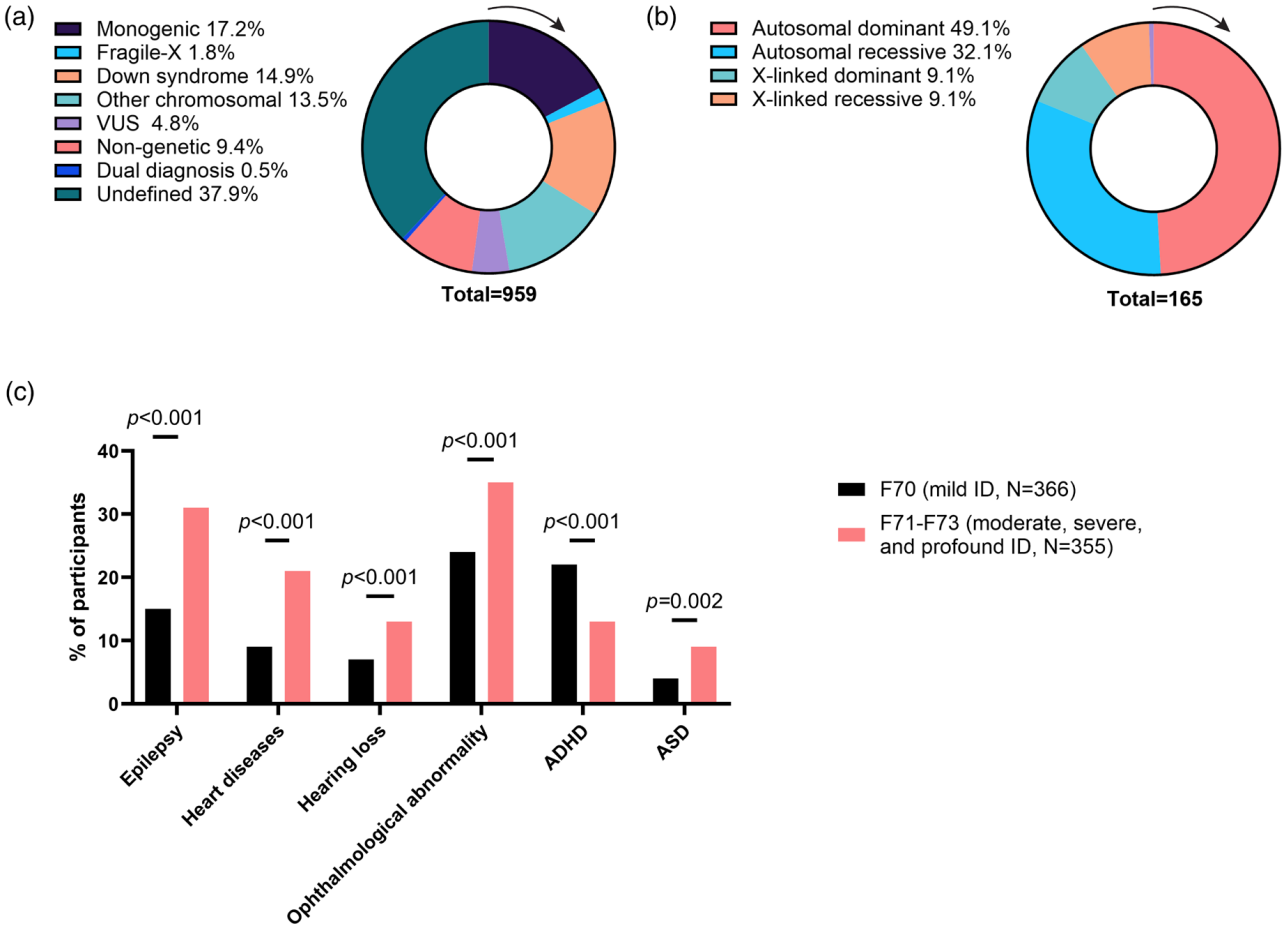
Aetiological factors and comorbidities in the cohort. (a) All aetiologies of intellectual disability (*n* = 959). (b) Modes of inheritance in monogenic intellectual disability (*n* = 165). (c) Epilepsy, congenital heart diseases, hearing loss, ophthalmological abnormalities, and ASD were more common in patients with moderate, severe, and profound intellectual disability than in those with mild intellectual disability. In contrast, ADHD was more common among patients with mild intellectual disability. Abbreviations: ADHD, attention‐deficit/hyperactivity disorder; ASD, autism spectrum disorder; VUS, variant of unknown significance.

Chromosomal abnormalities were identified in 272 out of 959 (28%) of the cohort. The most common chromosomal abnormality was 21‐trisomy, explaining 143 out of 959 (15%) of intellectual disability. There were four cases of trisomy 13 or 18. Other chromosomal abnormalities detected included 79 out of 959 (8%) deletions, 25 out of 959 (3%) duplications, 13 out of 959 (2%) complex aberrations, and 8 out of 959 (1%) other causative abnormalities.

Pathogenic or likely pathogenic sequence variants explaining the intellectual disability were identified in 165 out of 959 (17%) of patients. Pathogenic or likely pathogenic variants were consistent with an autosomal recessive mode of inheritance in 53 out of 165 (32%) cases (homozygous in 74% [*n* = 39] and compound heterozygous in 26% [*n* = 14]); autosomal dominant in 81 out of 165 (49%); X‐linked recessive in 15 out of 165 (9%); and X‐linked dominant in 15 out of 165 (9%) (Figure 1b). A total of 55 out of 81 (68%) of autosomal dominant and 10 out of 15 (67%) of X‐linked dominant variants were confirmed de novo. The most common causative genes were *GLDC* causing glycine encephalopathy (*n* = 11), *MECP2* causing Rett syndrome (*n* = 8), and *SLC17A5* causing infantile sialic acid storage disorder (*n* = 5). A variant of unknown significance (either sequence or chromosomal) was discovered for 46 out of 959 (5%) of the cohort. Classification of causative genes demonstrated heterogeneity in the biological processes and protein classes involved in the pathogenesis of intellectual disability (Figure S2). Autosomal recessive Finnish‐enriched variants causing the intellectual disability were identified in 39 patients in 19 distinct genes (Table S2). A Finnish‐enriched variant was identified in at least one allele in 39 out of 53 (74%) and in both alleles in 36 out of 53 (68%) of all patients with recessive inheritance. In addition, Prader–Willi syndrome was confirmed in seven patients and Angelman syndrome in fewer than three patients. Five patients were assessed as having a dual diagnosis explaining their intellectual disability.

Likely pathogenic or pathogenic gene variants were more common in the groups with epilepsy and ophthalmological abnormality comorbidity than in negative comparison groups (OR 2.07, 95% confidence interval [CI] 1.44–2.99; and OR 2.52, 95% CI 1.77–3.60). Chromosomal abnormalities were more common in the group with CHD comorbidity compared with the negative comparison group (OR 3.80, 95% CI 2.25–6.41) (Table S3).

We searched the treatable intellectual disability website (https://www.treatable‐id.org/) to identify genes associated with intellectual disability that have potential therapeutic targets and then reviewed the use of suggested treatment options in our cohort.^13^ Altogether, 18 out of 165 individuals (11%) had likely causative variants in seven genes: *AGA*, *ATP7A*, *CPS1*, *GLDC*, *IDS*, *MT‐TL1*, and *PDHA1*, all with potential therapeutic targets. Pharmacological and nutritional therapies and vitamin supplementation were used for conditions related to *CPS1*, *MT‐TL1*, and *PDHA1*. *GLDC* Finnish founder variants cause severe neonatal non‐ketotic hyperglycinaemia, leading to fatal outcomes during the perinatal period; therefore, no treatments were considered. For other patients, treatments were not considered owing to limited availability or insufficient evidence at the time of evaluation.

### Non‐genetic factors explaining intellectual disability

Ninety patients in the cohort (90 out of 959, 9%) had non‐genetic factors contributing to their intellectual disability. The most common non‐genetic causes included 19 patients with birth asphyxia, 16 born preterm, 15 with brain haemorrhage, eight with central nervous system infection (human herpesvirus, cytomegalovirus, or tick‐borne encephalitis), eight with ischemic brain injury, seven with brain malformations, and four with malignant brain tumours. Thirteen patients had other acquired causes probably explaining the intellectual disability including traumas and idiopathic causes. In all, 24 patients were born preterm: 12 extremely preterm (<28 gestational weeks), eight very preterm (28–32 weeks), four moderate to late preterm (32–37 weeks), or the preterm birth status was not determined. Eighteen patients had two or more non‐genetic factors presumably contributing to their intellectual disability. Non‐genetic aetiologies were more likely among patients with epilepsy (*n* = 49 out of 90, 54%; OR 3.83, 95% CI 2.44–6.04; *p* < 0.001) than among those without epilepsy (Table S3).

### Dual diagnosis

Five patients were found to have multiple likely causes explaining their intellectual disability, such as a combination of a chromosomal abnormality and a pathogenic gene variant, pathogenic gene variants in two independent known disease‐associated genes, or a genetic and non‐genetic cause.

### Severity of intellectual disability compared with aetiology and associated phenotypes

We compared the severity of intellectual disability between different aetiologies such as chromosomal abnormalities, likely causative gene variants, and acquired aetiologies (Tables 3 and S4). Chromosomal abnormalities and pathogenic and likely pathogenic gene variants were identified more often in patients with moderate, severe, and profound intellectual disability compared with mild intellectual disability (*p* < 0.001 and *p* = 0.011 respectively) (Table 3), but this difference was not significant in the diagnostic yield analysis (Table 4).

**TABLE 3:** Comparison between patients with mild (*n* = 366) and more severe (*n* = 355) forms of intellectual disability.

	F70 (mild)	F71–F73 (moderate, severe, and profound)	
	*n* (%)	*n* (%)	*p*
**Identified aetiological factors**			
**Monogenic aetiology** ^**a**^	47 (13)	73 (21)	0.007
Pathogenic or likely pathogenic variant	41 (11)	64 (18)	0.011
Fragile X syndrome	6 (2)	9 (3)	0.444
**Chromosomal aberrations**	62 (17)	126 (35)	<0.001***
21‐trisomy	5 (1)	85 (24)	
Deletions	34 (9)	27 (8)	
Duplications	14 (4)	7 (2)	
Other unbalanced rearrangements	9 (2)	7 (2)	
**Non‐genetic aetiology**	28 (8)	35 (10)	0.356
**Unknown**	226 (62)	120 (34)	<0.001***
**Associated phenotypes**			
**Epilepsy**	56 (15)	133 (31)	<0.001***
**Congenital heart diseases**	33 (9)	62 (21)	<0.001***
**Hearing loss**	25 (7)	53 (13)	<0.001***
**Ophthalmological abnormality**	88 (24)	148 (35)	<0.001***
**Attention‐deficit/hyperactivity disorder**	79 (22)	36 (13)	<0.001***
**Autism spectrum disorder**	13 (4)	33 (9)	0.002**
**Total number of patients**	366 (100)	355 (100)	

*Note*: Pathogenic or likely pathogenic gene variants and chromosomal aberrations were identified more frequently in patients with moderate, severe, and profound intellectual disability than in patients with mild intellectual disability (*p* = 0.011 and *p* < 0.001). Epilepsy, congenital heart diseases, hearing loss, ophthalmological abnormalities, and autism spectrum disorder were also more common in patients with severe forms of intellectual disability. In contrast, attention‐deficit/hyperactivity disorder was more common in patients with mild intellectual disability. Patients with dual diagnosis were not included in the analysis. Differences between intellectual disability groups were assessed using the χ^2^ test, except for the fragile X analysis, where Fisher's exact test was used due to the low number of patients.

^a^ Fragile X and pathogenic and likely pathogenic variants in the same analysis.

Bonferroni‐corrected threshold for statistical significance was 0.004; *p* < 0.001 marked with *** are highly significant; *p* < 0.01 marked with ** are very significant.

**TABLE 4:** Diagnostic yield of CMA and exome sequencing.

	CMA				Exome sequencing			
Group	*n* (CMA) (%)	*n* (%)	OR (95% CI)	*p*	*n* (exome sequencing) (%)	*n* (%)	OR (95% CI)	*p*
Mild intellectual disability (ICD‐10: F70), *n* = 366	241 (66)	51 (21)	1.0		65 (18)	27 (42)	1.0	
Moderate, severe, profound intellectual disability (ICD‐10: F71–F73), *n* = 355	173 (49)	32 (18)	0.81 (0.49–1.33)	0.656	70 (20)	33 (47)	1.24 (0.63–2.46)	0.529
Unspecified intellectual disability (ICD‐10: F79), *n* = 238	116 (49)	23 (20)	1.0 (0.58–1.78)	0.960	59 (25)	29 (49)	1.33 (0.63–2.78)	0.456
Total	530 (55)	106 (20)			194 (20)	89 (46)		

*Note*: Diagnostic yields were compared across intellectual disability severity levels, and OR values were estimated using logistic regression, adjusting for age and sex. CMA yield: 21% (mild intellectual disability) versus 18% (moderate–profound intellectual disability) (OR = 0.81, 95% CI 0.49–1.33, *p* = 0.656). Exome sequencing yield: 42% (mild intellectual disability) versus 47% (moderate–profound intellectual disability) (OR = 1.24, 95% CI 0.63–2.46, *p* = 0.529).

Abbreviations: CI, confidence interval; CMA, chromosomal microarray analysis; ICD‐10, International Classification of Diseases, 10th Revision; *n* (%), number of patients with a positive test result identifying the cause of intellectual disability in CMA or exome sequencing; *n* (CMA), number of patients who underwent CMA testing; *n* (exome sequencing), number of patients who underwent exome sequencing testing; OR, odds ratio.

Model fit: Nagelkerke *R* ^2^ (CMA) = 0.014; Nagelkerke *R* ^2^ (exome sequencing) = 0.006.

Finally, we compared the severity of intellectual disability and the presence of epilepsy, CHD, ophthalmological abnormalities, hearing loss, and neuropsychiatric diagnosis such as autism spectrum disorder and ADHD. ADHD was more common in patients with mild intellectual disability compared with severe forms (*p* < 0.001) (Tables 3 and S4), while epilepsy, CHD, hearing loss, and ophthalmological abnormalities and autism spectrum disorder were more common with more severe forms of intellectual disability.

### Diagnostic yield of genetic testing

The diagnostic yields of CMA, exome sequencing, and fragile X repeat expansion tests were 106 out of 530 (20%), 89 out of 194 (46%), and 17 out of 381 (4%) respectively. Table S5 presents clinical characteristics and comorbidities among patients who underwent CMA and exome sequencing.

We compared diagnostic yields between patients with mild intellectual disability and those with moderate, severe, or profound intellectual disability and found no significant differences (Table 4). For the fragile X repeat expansion test, the diagnostic yield was 6 out of 210 (3%) in patients with mild intellectual disability and 9 out of 123 (7%) in those with moderate, severe, or profound intellectual disability.

## DISCUSSION

We analysed the clinical characteristics and aetiological factors causing intellectual disability in a large, unselected Finnish clinical cohort. Chromosome abnormalities or gene variants explained 46% of intellectual disability in this cohort. Pathogenic or likely pathogenic gene variants in 106 genes and a multitude of different chromosome abnormalities were detected, demonstrating the genetic heterogeneity of intellectual disability. Approximately half of the cases of monogenic intellectual disability were caused by de novo autosomal dominant variants.

Variants enriched in the Finnish population accounted for 39 out of 165 (24%) of all pathogenic or likely pathogenic variants, reflecting the population's history of isolation, genetic drift, repeated bottleneck events, and subsequent population expansion, which contributed to the accumulation of unique variants.^14^ Our cohort included 33 patients whose intellectual disability was caused by diseases belonging to the Finnish disease heritage, such as non‐ketotic hyperglycinaemia, autosomal recessive sialuria (Finnish type), and aspartylglucosuria.^14^ The high prevalence of these founder variants enables the application of targeted Sanger sequencing to identify known pathogenic variants in Finnish patients presenting with phenotypes typical of Finnish disease heritage disorders. A similar genetic testing strategy may be applicable in other founder populations; however, it cannot be generalized to genetically diverse populations, where multiple pathogenic variants may underlie the same disease. This cohort is representative of the Finnish population, as 97% of the individuals in it were of Finnish ancestry.

Notably, 18 of 165 patients (11%) with a pathogenic or likely pathogenic gene variant had a variant in an intellectual disability gene with a potential therapeutic target.^13^ Pharmacological therapies are recommended for conditions associated with *ATP7A*, *CPS1*, and *GLDC* genes, such as sodium phenylbutyrate or sodium benzoate to prevent acute metabolic decompensation in *CPS1*‐related disorders. Nutritional interventions are advised for disorders linked to *CPS1*, *MT‐TL1*, and *PDHA1*, for example a ketogenic diet to improve cognitive or psychomotor development in *PDHA1*‐related disorders. Vitamin and trace element supplementation, such as thiamine, is recommended for pathogenic *PDHA1* variants to prevent or slow clinical deterioration and improve seizure control. Recently, a customized lipid nanoparticle‐delivered base‐editing therapy was administered to an infant with severe CPS1 deficiency.^15^ Despite advances in orphan drug development, effective treatments remain limited. For potentially treatable conditions, it is essential to regularly review treatment guidelines and monitor ongoing clinical trials.

Interestingly, the diagnostic yield of CMA was similar between patients with mild intellectual disability and those with moderate to profound intellectual disability. Similarly, the difference in exome sequencing diagnostic yield between these groups was also modest (Table 4). Since only a proportion of patients underwent genetic testing, this result may reflect that clinicians were more likely to offer testing to individuals who exhibited features commonly associated with a positive result, such as epilepsy or dysmorphic features. Conversely, a growing number of genes and chromosomal abnormalities have been associated with mild intellectual disability and are known to exhibit reduced penetrance, supporting the use of exome sequencing and CMA in individuals with mild intellectual disability. We included structural variants with known low penetrance, such as 15q11.2 deletions, as likely causal factors, which may have increased the diagnostic yield. The diagnostic yield of exome sequencing in our study was relatively high (*n* = 89 out of 194, 46%) compared with previous estimates of 36% in neurodevelopmental disorders.^16^ This is probably attributable to the use of exome sequencing as a second‐tier test during the study period for patients with a high clinical suspicion of a genetic syndrome. The diagnostic yield of fragile X test was relatively low, supporting the use of fragile X testing as a second‐tier test.^17^

In this study, autosomal recessive variants comprised 32% of all likely causal variants. In contrast, recent studies in Finnish and British populations have suggested that the proportion of intellectual disability cases attributable to recessive coding variants is only 2% to 4%.^18^, ^19^ The true proportion may be higher owing to ascertainment bias in cohorts recruiting only individuals with intellectual disability of unknown aetiology. This bias could reduce the number of recessive diagnoses as some diseases, such as metabolic disorders, are probably diagnosed in clinical practice and therefore not recruited to research projects.^18^, ^19^ Population‐based cohort studies are required to establish the contribution of recessive coding variants in intellectual disability.

Our study has several limitations. As a retrospective registry‐based study, a variety of genetic testing approaches were used; and, in several patients, limited genetic testing was performed. In addition, some patients were tested for targeted pathogenic familial variants (*n* = 9) or known Finnish founder variants (*n* = 61), and some patients may have been related, potentially increasing the proportion of pathogenic recessive variants in our cohort. A substantial number of individuals with intellectual disability of unknown aetiology had not undergone CMA (118 out of 410, 29%) or exome sequencing (329 out of 410, 80%). It is likely that some of these individuals had a genetic aetiology explaining their intellectual disability. Especially among those with mild intellectual disability, the aetiology remained unknown (*p* < 0.001). In addition, detailed phenotypic information, such as assessment of the severity of intellectual disability, was missing for a considerable proportion of the cohort, which included children at different ages and phases of the intellectual disability trajectory. In the future, prospective population‐based studies will provide more precise knowledge of the genetic aetiology and natural history of intellectual disability.

The implementation of next‐generation sequencing in standard clinical practice has resulted in a significant increase in the diagnostic yield of intellectual disability. Elucidation of molecular defects causing intellectual disability allows the development of tailored therapeutic strategies. A potential therapeutic intervention was available for 18 of 165 patients (11%) with a pathogenic or likely pathogenic variant in this cohort.^13^ The proportion of potentially treatable cases of intellectual disability is likely to increase in the future.

In our cohort, the proportion of patients with Down syndrome was higher than previously reported estimates.^20^ This may reflect regional attitudes towards 21‐trisomy screening and elective pregnancy termination in Northern Finland. The elevated proportion could also have been influenced by the study design, as patients were included if they visited our hospital between 2017 and 2021. Patients with Down syndrome generally require regular hospital follow‐up, whereas some individuals with mild intellectual disability may remain undiagnosed or may not need hospital‐based follow‐up.

In conclusion, this study demonstrates the complex genetic landscape of intellectual disability in the Finnish founder population. While our results on differences between mild and severe forms of intellectual disability can be generalized to other populations, the unique distribution of pathogenic gene variants is specific to the Finnish population. Interestingly, diagnostic yield analysis did not show significant differences between mild and more severe forms of intellectual disability. In contrast, there were phenotypic differences between mild and severe intellectual disability, as somatic comorbidities such as epilepsy were more common in severe forms of intellectual disability while ADHD was more common in mild intellectual disability. Our data provide justifications for genetic studies in mild intellectual disability and a focus for future research into the causes, natural history, and novel treatment options of intellectual disability.

## FUNDING INFORMATION

This study was funded by Research Council of Finland (grant numbers 338446, 331436, and 356676), the Finnish Brain Foundation (grant number 20230066), Terttu Foundation, the Finnish Medical Foundation, the Sigrid Juselius Foundation, the Foundation for Paediatric Research, Finland, the Alma and K. A. Snellman Foundation, Oulu, Finland, the Competitive State Funding for Health Research for the Wellbeing Services County of North Ostrobothnia, and the Medical Research Center Oulu.

## CONFLICT OF INTEREST STATEMENT

The authors have stated that they had no interests that might be perceived as posing a conflict or bias.

## Supporting information


**Figure S1:** Flowchart of the study population.

**Figure S2:** Classification of genes with pathogenic and likely pathogenic variants.

**Table S1:** Pathogenic or likely pathogenic chromosomal aberrations.

**Table S2:** Autosomal recessive pathogenic or likely pathogenic variants enriched in the Finnish population identified in this study.

**Table S3:** Comparison between genetic etiology and associated comorbidities.

**Table S4:** The number of patients with mild, moderate, severe, profound, and unspecified intellectual disability and the numbers of patients in each category with different etiological factors and different associated phenotypes.

**Table S5:** Clinical characteristics and comorbidities among patients who underwent CMA and ES testing.

## Data Availability

All anonymized data generated during this study are included in this published article and its supplementary information files. The patients of this study did not give written consent for their data to be shared publicly; so, because of the sensitive nature of the research, supporting data are not available.

## References

[1] Olusanya BO , Wright SM , Nair MKC , Boo NY , Halpern R , Kuper H , et al. Global burden of childhood epilepsy, intellectual disability, and sensory impairments. Pediatrics. 2020; 146(1). 10.1542/peds.2019-2623 PMC7613313 32554521

[2] Ilyas M , Mir A , Efthymiou S , Houlden H . The genetics of intellectual disability: Advancing technology and gene editing. F1000Res. 2020; 9(22). 10.12688/f1000research.16315.1 PMC6966773 31984132

[3] Jansen S , Vissers LELM , de Vries BBA . The Genetics of Intellectual Disability. Brain Sci. 2023; 13(2). 10.3390/brainsci13020231 PMC9953898 36831774

[4] Järvelä I , Määttä T , Acharya A , Leppälä J , Jhangiani SN , Arvio M , et al. Exome sequencing reveals predominantly de novo variants in disorders with intellectual disability (ID) in the founder population of Finland. Hum Genet 2021; 140(7): 1011–29. 10.1007/s00439-021-02268-1 PMC8197721 33710394

[5] Hu H , Kahrizi K , Musante L , Fattahi Z , Herwig R , Hosseini M , et al. Genetics of intellectual disability in consanguineous families. Mol Psychiatry. 2019; 24(7): 1027–39. 10.1038/s41380-017-0012-2 29302074

[6] Kurki MI , Saarentaus E , Pietiläinen O , Gormley P , Lal D , Kerminen S , et al. Contribution of rare and common variants to intellectual disability in a sub‐isolate of Northern Finland. Nat Commun 2019; 10(1). 10.1038/s41467-018-08262-y PMC6345990 30679432

[7] Reichenberg A , Cederlöf M , McMillan A , Trzaskowski M , Kapara O , Fruchter E , et al. Discontinuity in the genetic and environmental causes of the intellectual disability spectrum. Proc Natl Acad Sci U S A. 2016; 113(4): 1098–103. 10.1073/pnas.1508093112 PMC4743770 26711998

[8] Westerinen H , Kaski M , Virta LJ , Kautiainen H , Pitkälä KH , Iivanainen M . The nationwide register‐based prevalence of intellectual disability during childhood and adolescence. Intellect Disabil Res. 2017; 61(8): 802–9. 10.1111/jir.12351 28090701

[9] World Health Organization . Preterm birth. World Health Organization Internet sites. 2023. https://www.who.int/news‐room/fact‐sheets/detail/preterm‐birth (accessed 1 Oct 2024).

[10] Richards S , Aziz N , Bale S , Bick D , Das S , Gastier‐Foster J , et al. Standards and guidelines for the interpretation of sequence variants: A joint consensus recommendation of the American College of Medical Genetics and Genomics and the Association for Molecular Pathology. Genet Med. 2015; 17(5): 405–24. 10.1038/gim.2015.30 PMC4544753 25741868

[11] Riggs ER , Andersen EF , Cherry AM , Kantarci S , Kearney H , Patel A , et al. Technical standards for the interpretation and reporting of constitutional copy‐number variants: a joint consensus recommendation of the American College of Medical Genetics and Genomics (ACMG) and the Clinical Genome Resource (ClinGen). Genet Med. 2020; 22(2): 245–57. 10.1038/s41436-019-0686-8 PMC7313390 31690835

[12] Chen S , Francioli LC , Goodrich JK , Collins RL , Kanai M , Wang Q , et al. A genomic mutational constraint map using variation in 76,156 human genomes. Nature. 2024; 625(7993): 92–100. 10.1038/s41586-023-06045-0 PMC11629659 38057664

[13] Hoytema van Konijnenburg EMM , Wortmann SB , Koelewijn MJ , Tseng LA , Houben R , Stöckler‐Ipsiroglu S , et al. Treatable inherited metabolic disorders causing intellectual disability: 2021 review and digital app. Orphanet J Rare Dis. 2021; 16(1). 10.1186/s13023-021-01727-2 PMC8042729 33845862

[14] Uusimaa J , Kettunen J , Varilo T , Järvelä I , Kallijärvi J , Kääriäinen H , et al. The Finnish genetic heritage in 2022‐from diagnosis to translational research. Dis Model Mech. 2022; 15(10). 10.1242/dmm.049490 PMC9637267 36285626

[15] Musunuru K , Grandinette SA , Wang X , Hudson TR , Briseno K , Berry A , et al. Patient‐Specific In Vivo Gene Editing to Treat a Rare Genetic Disease. N Engl J Med. 2025; 392(22): 2235–43. 10.1056/NEJMoa2504747 PMC12713542 40373211

[16] Srivastava S , Love‐Nichols JA , Dies KA , Ledbetter DH , Martin CL , Firth H , et al. Meta‐analysis and multidisciplinary consensus statement: exome sequencing is a first‐tier clinical diagnostic test for individuals with neurodevelopmental disorders. Genet Med. 2019; 21(11): 2413–21. 10.1038/s41436-019-0554-6 PMC6831729 31182824

[17] Borch LA , Parboosingh J , Thomas MA , Veale P . Re‐evaluating the first‐tier status of fragile X testing in neurodevelopmental disorders. Genet Med. 2020; 22(6): 1036–9. 10.1038/s41436-020-0773-x 32152462

[18] Martin HC , Jones WD , McIntyre R , Sanchez‐Andrade G , Sanderson M , Stephenson JD , et al. Quantifying the contribution of recessive coding variation to developmental disorders. Science. 2018; 362(6419): 1161–4. 10.1126/science.aar6731 PMC6726470 30409806

[19] Urpa L , Kurki MI , Rahikkala E , Hämäläinen E , Salomaa V , Suvisaari J , et al. Evidence for the additivity of rare and common variant burden throughout the spectrum of intellectual disability. Eur J Hum Genet. 2024; 32(5): 576–83. 10.1038/s41431-024-01581-3 PMC11061114 38467730

[20] Vissers LELM , Gilissen C , Veltman JA . Genetic studies in intellectual disability and related disorders. Nat Rev Genet. 2016; 17(1): 9–18. 10.1038/nrg3999 26503795

