# Supplementary material for: Clinical and genetic characterization of intellectual disability
Source: Dev Med Child Neurol. 2026 Mar 17;68(11):1601–9. doi: 10.1111/dmcn.70252 (PMC13649302; doi:10.1111/dmcn.70252)
Supplement: Supplementary file 1 [file DMCN-68-1601-s001.pdf]

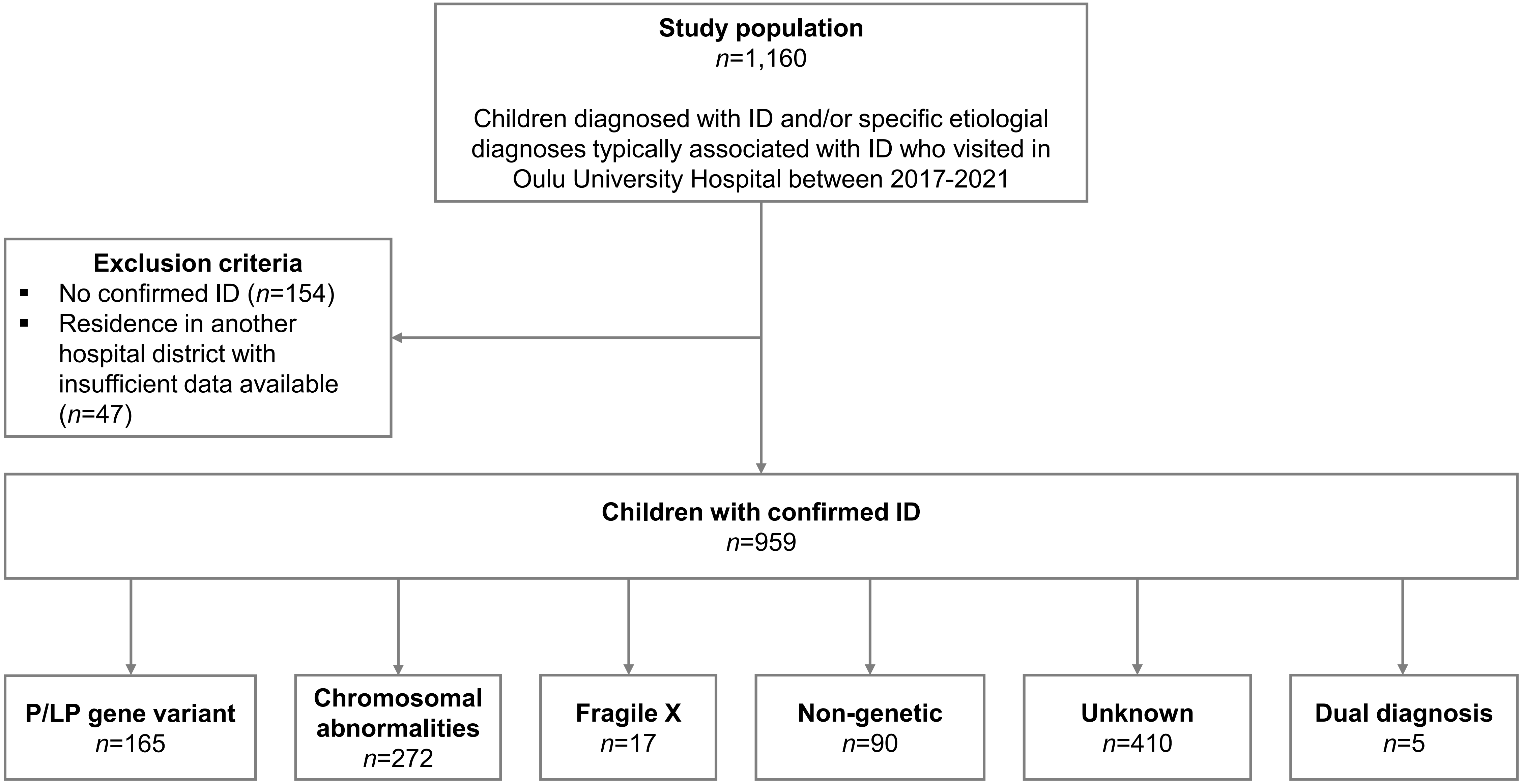
Study population
n=1,160
Children diagnosed with ID and/or specific etiologial diagnoses typically associated with ID who visited in Oulu University Hospital between 2017-2021
Exclusion criteria
No confirmed ID (n=154)
Residence in another hospital district with insufficient data available (n=47)
Children with confirmed ID
n=959
P/LP gene variant
n=165
Chromosomal abnormalities
n=272
Fragile X
n=17
Non-genetic
n=90
Unknown
n=410
Dual diagnosis
n=5
