# Supplementary material for: Clinical and genetic characterization of intellectual disability
Source: Dev Med Child Neurol. 2026 Mar 17;68(11):1601–9. doi: 10.1111/dmcn.70252 (PMC13649302; doi:10.1111/dmcn.70252)
Supplement: Supplementary file 2 [file DMCN-68-1601-s002.docx]

**Table S5. Clinical characteristics and comorbidities among patients who underwent CMA and ES testing.**

| ***Severity of ID*** | **CMA** | **ES** |
| --- | --- | --- |
| Mild | 241 (45.5%) | 65 (33.5%) |
| Moderate | 76 (14.3%) | 32 (16.5%) |
| Severe | 65 (12.3%) | 21(10.8%) |
| Profound | 32 (6.0%) | 17 (8.8%) |
| Unspecified | 116 (21.9%) | 59 (30.4%) |
| ***Comorbidities*** |  |  |
| Epilepsy | 140 (26.4%) | 82 (42.3%) |
| CHDs | 48 (9.1%) | 19 (9.8%) |
| Hearing loss | 46 (8.7%) | 14 (7.2%) |
| Ophthalmological abnormality | 156 (29.4%) | 75 (38.7%) |
| ***Neuropsychiatric disorder*** |  |  |
| ADHD | 85 (16.0%) | 20 (10.3%) |
| ASD | 37 (7.0%) | 16 (8.2%) |
