# Supplementary material for: Clinical and genetic characterization of intellectual disability
Source: Dev Med Child Neurol. 2026 Mar 17;68(11):1601–9. doi: 10.1111/dmcn.70252 (PMC13649302; doi:10.1111/dmcn.70252)
Supplement: Supplementary file 3 [file DMCN-68-1601-s003.docx]

**Table S2**. Autosomal recessive pathogenic or likely pathogenic variants enriched in the Finnish population identified in this study. Several autosomal recessively inherited ID associated genes were highly enriched in the Finnish population. The identified variants were classified using ACMG criteria.^2^ Variants’ population frequencies were obtained from the Genome Aggregation Database (gnomAD v.4.1.0).^3^

| **Gene** | **Transcript** | **Variant** | **Amino Acid changes** | **ACMG criteria** | **rs number** | **Inheritance** | **Total allele freq in GnomAD v.4.1.0** | **Allele freq in Finns** | **Allele freq in non-Finnish Europeans** | **Allele freq in Finns/Allele freq in non-Finnish Europeans** |
| --- | --- | --- | --- | --- | --- | --- | --- | --- | --- | --- |
| AAAS | NM_015665.6 | c.787T>C | p.(Ser263Pro) | P | rs121918550 | AR | 0.00007622 | 0.0005468 | 0.00007034 | 7.8 |
| AGA | NM_000027.4 | c.488G>C | p.(Cys163Ser) | P | rs121964904 | AR | 0.0003464 | 0.007673 | 0.00003165 | 242.4 |
| BBS1 | NM_024649.5 | c.724-1G>C | p.? | P | rs748523268 | AR | 0.00004214 | 0.000937 | 0.000005086 | 184.2 |
| CLN3 | NM_001042432.2 | c.1001G>A | p.(Arg334His) | P | rs386833695 | AR | 0.00002293 | 0.000172 | 0.00002034 | 8.5 |
| CRADD | NM_003805.5 | c.509G>A | p.(Arg170His) | LP | rs141179774 | AR | 0.0002739 | 0.005861 | 0.00004154 | 141.1 |
| CTC1 | NM_025099.6 | c.680C>T | p.Ala227Val | LP | rs199473673 | AR | 0.00004957 | 0.001235 | 8.475E-7 | 1457.2 |
| CTC1 | NM_025099.6 | c.3019del | p.Leu1007Cysfs*62 | P | rs199473680 | AR | 0.0001612 | 0.0008281 | 0.0001688 | 4.9 |
| DIAPH1 | NM_005219.5 | c.684+1G>A | p.? | P | rs766545876 | AR | 0.00003285 | 0.000781 | 0.000002544 | 307.0 |
| GLDC | NM_000170.3 | c.1691G>T | p.(Ser564Ile) | P | rs121964974 | AR | 0.00007444 | 0.001858 | 8.487E-7 | 2189.2 |
| GLDC | NM_000170.3 | c.2281G>A | p.(Gly761Arg) | P | rs386833549 | AR | 0.0000583 | 0.001296 | 0.000005933 | 218.4 |
| GNPTAB | NM_024312.5 | c.1581del | p.(Cys528Valfs*19) | P | rs36007394 | AR | 0.0001152 | 0.002842 | 0.000001695 | 1676.7 |
| MTRFR | NM_152269.5 | c.43C>T | p.(Arg15*) | P | rs757148270 | AR | 0.00005266 | 0.001171 | 0.000007627 | 153.5 |
| OAT | NM_000274.4 | c.1205T>C | p.(Leu402Pro) | P | rs121965043 | AR | 0.0001109 | 0.002562 | 0.000009322 | 274.8 |
| POMGNT1 | NM_017739.4 | c.879+5G>T | p.? | P | rs386834038 | AR | 0.000001858 | 0.00004684 | 0 | ∞ |
| POMGNT1 | NM_017739.4 | c.1539+1G>A | p.? | P | rs138642840 | AR | 0.0003532 | 0.002155 | 0.0003298 | 6.5 |
| PPT1 | NM_000310.4 | c.364A>T | p.(Arg122Trp) | P | rs137852695 | AR | 0.0003267 | 0.006907 | 0.00004494 | 153.7 |
| PRRT2 | NM_145239.3 | c.649dup | p.(Arg217Profs*8) | P | rs587778771 | AR | 0.0006636 | 0.003776 | 0.000513 | 7.4 |
| SLC17A5 | NM_012434.5 | c.115C>T | p.(Arg39Cys) | P | rs80338794 | AR | 0.0003643 | 0.004561 | 0.0002305 | 19.8 |
| SUCLA2 | NM_003850.3 | c.998A>G | p.(Asp333Gly) | LP | rs140963290 | AR | 0.00005515 | 0.001078 | 0.00001102 | 97.8 |
| TWNK | NM_021830.5 | c.1523A>G | p.(Tyr508Cys) | P | rs80356540 | AR | 0.0001183 | 0.002655 | 0.0000161 | 164.9 |
| VPS13B | NM_152564.5 | c.3348_3349del | p.(Cys1117Phefs*8) | P | rs180177327 | AR | 0.0001109 | 0.002686 | 0.000001695 | 1584.7 |
| ZNHIT3 | NM_004773.4 | c.92C>T | p.(Ser31Leu) | P | rs148890852 | AR | 0.0001967 | 0.004411 | 0.00001865 | 236.5 |

Abbreviations: P, pathogenic; LP, likely pathogenic; AR, autosomal recessive; freq, frequency

**References**

1	Thomas PD, Ebert D, Muruganujan A, Mushayahama T, Albou LP, Mi H. PANTHER: Making genome-scale phylogenetics accessible to all. *Protein Sci.* 2022; **31**(1): 8–22.

2	Richards S, Aziz N, Bale S, Bick D, Das S, Gastier-Foster J, et al. Standards and guidelines for the interpretation of sequence variants: A joint consensus recommendation of the American College of Medical Genetics and Genomics and the Association for Molecular Pathology. *Genet Med.* 2015; **17**(5): 405–24.

3	Chen S, Francioli LC, Goodrich JK, Collins RL, Kanai M, Wang Q, et al. A genomic mutational constraint map using variation in 76,156 human genomes. *Nature.* 2024; **625**(7993): 92–100.
