# Supplementary material for: Clinical and genetic characterization of intellectual disability
Source: Dev Med Child Neurol. 2026 Mar 17;68(11):1601–9. doi: 10.1111/dmcn.70252 (PMC13649302; doi:10.1111/dmcn.70252)
Supplement: Supplementary file 4 [file DMCN-68-1601-s005.docx]

**Table S3.** Comparison between genetic etiology and associated comorbidities. Logistic regression was used to assess whether pathogenic gene variants or chromosomal abnormalities were more frequently detected in patients with comorbidities. Differences between ID groups were evaluated using logistic regression, with epilepsy, hearing loss, ophthalmological abnormalities, CHD, ADHD, and ASD as dichotomous outcome variables. Pathogenic gene variants were more likely among patients with epilepsy (OR 2.07, 95 % CI 1.44–2.99) and ophthalmological comorbidities (OR 2.52, 95 % CI 1.77–3.60) compared with those without these comorbidities. Chromosomal abnormalities were more likely among patients with CHD compared with those without CHD (OR 3.80, 95 % CI 2.25–6.41). Bonferroni corrected threshold for statistical significance was 0.003, p-values marked with † remained significant after correction.

|  | **N** | **P/LP variants (*n* = 165)** | **OR** | **95 % CI** | ***p*-value** | **Nagelkerke R²** | **N** | **Chromosomal abnormalities* (*n* = 125)** | **OR** | **95 % CI** | ***p*-value** | **Nagelkerke R^2^** |
| --- | --- | --- | --- | --- | --- | --- | --- | --- | --- | --- | --- | --- |
| **ID without epilepsy** | 473 | 96 | 1.00 |  |  | 0.039 | 477 | 92 | 1.00 |  |  | 0.013 |
| **ID with epilepsy** | 174 | 69 | 2.07 | 1.44–2.99 | < 0.001† |  | 210 | 33 | 0.74 | 0.48–1.15 | 0.178 |  |
| **ID without hearing loss** | 592 | 147 | 1.00 |  |  | 0.012 | 632 | 107 | 1.00 |  |  | 0.020 |
| **ID with hearing loss** | 55 | 18 | 1.36 | 0.77–2.40 | 0.285 |  | 55 | 18 | 1.99 | 1.13–3.50 | 0.018 |  |
| **ID without ophthalmological abnormality** | 468 | 85 | 1.00 |  |  | 0.059 | 474 | 79 | 1.00 |  |  | 0.010 |
| **ID with ophthalmological abnormality** | 179 | 80 | 2.52 | 1.77–3.60 | < 0.001† |  | 213 | 46 | 1.20 | 0.80–1.80 | 0.373 |  |
| **ID without CHD** | 586 | 153 | 1.00 |  |  | 0.012 | 641 | 98 | 1.00 |  |  | 0.056 |
| **ID with CHD** | 61 | 12 | 0.77 | 0.40–1.47 | 0.422 |  | 46 | 27 | 3.80 | 2.25–6.41 | < 0.001† |  |
| **ID without ADHD** | 536 | 156 | 1.00 |  |  | 0.039 | 583 | 109 | 1.00 |  |  | 0.009 |
| **ID with ADHD** | 111 | 9 | 0.30 | 0.15–0.60 | < 0.001† |  | 104 | 16 | 0.88 | 0.50–1.57 | 0.671 |  |
| **ID without ASD** | 599 | 159 | 1.00 |  |  | 0.016 | 636 | 122 | 1.00 |  |  | 0.019 |
| **ID with ASD** | 48 | 6 | 0.50 | 0.21–1.20 | 0.120 |  | 51 | 3 | 0.31 | 0.10–1.02 | 0.055 |  |

Abbreviations: P, pathogenic; LP, likely pathogenic; OR, odds ratio; CI, confidence interval

*Patients with 21-trisomy (*n* = 143) and other trisomies (*n* = 4) were excluded from the analysis, as their inclusion would have introduced a significant confounding factor. In this cohort, 21-trisomy was associated with CHD (*n*=62/143, 43%; OR 7.73, 95% CI 5.12–11.66; *p*<0.001†; Nagelkerke R^2^ = 0.159) and hearing loss (*n*=33/143, 23%; OR 3.18, 95% CI 2.00–5.06; *p*<0.001†; Nagelkerke R^2^ = 0.040).

Non-genetic etiologies were more likely among patients with epilepsy (*n*=49/90, 54%; OR 3.83, 95% CI 2.44–6.04; *p*<0.001†; Nagelkerke R^2^ = 0.082) than among those without epilepsy. There was a trend toward non‑genetic etiologies being more common among patients with ophthalmological abnormalities (n=40/90, 44%; OR 1.80, 95% CI 1.15–2.80; *p*=0.01; Nagelkerke R^2^ = 0.023) than among those without ophthalmological abnormalities, but this association was not significant after Bonferroni correction.
