# Supplementary material for: Clinical and genetic characterization of intellectual disability
Source: Dev Med Child Neurol. 2026 Mar 17;68(11):1601–9. doi: 10.1111/dmcn.70252 (PMC13649302; doi:10.1111/dmcn.70252)
Supplement: Supplementary file 5 [file DMCN-68-1601-s006.docx]

**Figure S2. Classification of genes with pathogenic and likely pathogenic variants.** Likely causative genes (*n*=105) in the cohort were classified using Panther classification according to the molecular function (A), biological process (B) and protein class (C) of the gene.^1^
