# Supplementary material for: Clinical and genetic characterization of intellectual disability
Source: Dev Med Child Neurol. 2026 Mar 17;68(11):1601–9. doi: 10.1111/dmcn.70252 (PMC13649302; doi:10.1111/dmcn.70252)
Supplement: Supplementary file 6 [file DMCN-68-1601-s007.docx]

**Table S4.** The number of patients with mild (F70), moderate (F71), severe (F72), profound (F73) and unspecified (F79, F78) ID and the numbers of patients in each category with different etiological factors and different associated phenotypes. The differences between ID groups were calculated using chi-squared test. Pathogenic and likely pathogenic gene variants and chromosomal aberrations were identified more frequently in patients with severe forms of ID (*p*<0.001 and *p*<0.001). Epilepsy, CHD, hearing loss, ophthalmological abnormality, and ASD were associated with more severe forms of ID while ADHD was associated with mild ID (*p*<0.001). *Patients with Fragile X syndrome (*n*=17) or dual diagnosis (*n*=5) were not included in the analysis due to the low number of cases. **Unspecified forms of IDs were excluded from the analysis of severity. Bonferroni corrected threshold for statistical significance was 0.005; p-values marked with † remained significant after correction.

|  | **F70 (mild)** | **F71 (moderate)** | **F72 (severe)** | **F73 (profound)** | **F79+NA (unspecified)**** | ***p*-value** |  |
| --- | --- | --- | --- | --- | --- | --- | --- |
|  | ***n* (%)** | ***n* (%)** | ***n* (%)** | ***n* (%)** | ***n* (%)** |  |  |
| ***Identified etiological factors*~~*~~** |  |  |  |  |  |  |  |
| **Pathogenic or likely pathogenic variant** | **41 (11)** | **28 (16)** | **17 (14)** | **19 (32)** | **60 (25)** | **< 0.001**† |  |
| **Chromosomal aberrations** | **62 (17)** | **63 (36)** | **49 (41)** | **14 (23)** | **84 (35)** | **< 0.001**† |  |
| **Non-genetic etiology** | **28 (8)** | **16 (9)** | **9 (8)** | **10 (17)** | **27 (11)** | **0.136** |  |
| **Unknown** | **226 (62)** | **60 (34)** | **43 (36)** | **17 (28)** | **64 (27)** | **< 0.001**† |  |
| **Total number of patients** | **366 (100)** | **175 (100)** | **120 (100)** | **60 (100)** | **238 (100)** |  |  |
|  |  |  |  |  |  |  |  |
| ***Associated phenotypes*** |  |  |  |  |  |  |  |
| **Epilepsy** | **56 (15)** | **55 (31)** | **35 (29)** | **43 (72)** | **65 (27)** | **< 0.001**† |  |
| **CHDs** | **33 (9)** | **36 (21)** | **20 (17)** | **6 (10)** | **42 (18)** | **0.001**† |  |
| **Hearing loss** | **25 (7)** | **23 (13)** | **23 (19)** | **7 (12)** | **28 (12)** | **0.001**† |  |
| **Ophthalmological abnormality** | **88 (24)** | **61 (35)** | **48 (40)** | **39 (65)** | **67 (28)** | **< 0.001**† |  |
| **ADHD** | **79 (22)** | **23 (13)** | **13 (11)** | **0 (0)** | **11 (5)** | **< 0.001**† |  |
| **ASD** | **13 (4)** | **9 (5)** | **15 (13)** | **9 (15)** | **8 (3)** | **< 0.001**† |  |
| **Total number of patients** | **366 (100)** | **175 (100)** | **120 (100)** | **60 (100)** | **238 (100)** |  |  |
|  |  |  |  |  |  |  |  |
|  |  |  |  |  |  |  |  |
